# Association between perceived neighborhood environment, sedentary behavior, walking, and moderate-to-vigorous physical activity and frailty: an isotemporal substitution model

**DOI:** 10.1186/s12877-025-06200-4

**Published:** 2025-07-29

**Authors:** Hee-kyoung Nam, Chang Won Won, Miji Kim, Sung-il Cho

**Affiliations:** 1 Department of Public Health Science, Graduate School of Public Health, Seoul National University, 1 Gwanak-ro, Gwanak-gu, Seoul, Republic of Korea; 2 Department of Family Medicine, Elderly Frailty Research Center, College of Medicine, Kyung Hee University, Seoul, Republic of Korea; 3 Department of Health Sciences and Technology, College of Medicine, Kyung Hee University, Seoul, Republic of Korea; 4 Institute of Health and Environment, Seoul National University, Seoul, Republic of Korea

**Keywords:** Frailty, Physical activity, IPAQ-E, Built environment, KFACS

## Abstract

**Introduction:**

With the rapid increases of older population and growing demand for longevity worldwide, frailty has become a major hurdle to sustaining healthy aging. As residential areas are the primary domains of mobility for older adults, the neighborhood environment is a crucial factor for their daily living and physical activity. This study aims to investigate whether replacing sedentary behavior with physical activity and having a supportive neighborhood environment are associated with frailty status in older adults.

**Methods:**

A cross-sectional analysis was conducted with 2,650 participants aged 70–84 years from the Korean Frailty and Aging Cohort Study (KFACS). The main explanatory variables included self-reported physical activity, sedentary behavior (SB), and perceived neighborhood environment. Frailty as the response variable was defined using the Fried frailty phenotype. Multinomial regression was performed to analyze the outcome. The Isotemporal Substitution Model (ISM) was applied to examine the effects of replacing sedentary behavior with physical activity.

**Results:**

The frailty status of participants was categorized as 45.3% robust, 47.3% pre-frail, and 7.6% frail. Accessibility factor was associated with a decreased risk of being pre-frail (odds ratio (OR): 0.750, 95% CI: 0.673–0.836) and frail (OR: 0.654, 95% CI: 0.541–0.789) compared to being robust. According to ISM, substituting 10 min of SB with any type of physical activity was associated with a reduced risk of pre-frailty [if 10 min of SB was replaced by 10 min of walking (OR: 0.972, CI: 0.960–0.985)] and frailty [if 10 min of SB was replaced by MVPA (OR: 0.877, CI: 0.836–0.921); or by walking (OR: 0.852, CI: 0.814–0.891)].

**Conclusions:**

Replacing SB with walking and improving neighborhood accessibility were significantly associated with reduced risk of being pre-frail or frail. These findings highlight the importance of considering these factors when designing age-friendly environments for older adults.

**Supplementary Information:**

The online version contains supplementary material available at 10.1186/s12877-025-06200-4.

## Introduction

With the rapid increases of older population and growing demand for longevity worldwide, frailty has become a major hurdle to sustaining healthy aging. Frailty, characterized by a progressive decline in physiological capacity and functional ability, is influenced by a complex interaction of physical, social, and environmental determinants [1, 2]. For instance, biological age, polypharmacy, physical inactivity, living alone, female sex, low socio-economic status, malnutrition, and a lack of age-friendly environments are known to be key determinants of an increased risk of frailty [3, 4]. Given that frailty is associated with fall injuries [5], all cause of mortality [6] and is a leading cause of death in later life [7], its prevention is of paramount importance. Moreover, as Korea is projected to undergo a rapid demographic shift, with the population of adults over 65 expected to increase from 18% in 2022 to 37.3% in 2045 [8], it is crucial to enhance the monitoring of aging-related diseases to implement effective interventions in a timely manner.

When considering the high prevalence of frailty (12%) and pre-frailty (46%) globally [9], prompt intervention, such as physical activity for older adults is essential. Such interventions are particularly recommended in line with guidelines that emphasize reducing sedentary behavior (SB). According to the World Health Organization (WHO) guidelines, adults over 65 years are advised to engage in 150–300 min of moderate-intensity or 75–150 min of vigorous-intensity physical activity weekly [10]. The International Conference of Frailty and Sarcopenia Research (ICFSR) task force recommends that older adults with frailty participate in multi-component physical activities to enhance overall physical abilities, such as gait speed and muscle strength, which subsequently improve balance [11]. Additionally, the Integrated Care for Older People (ICOPE) guidelines advocate for multimodal exercises, including strength resistance, flexibility, and aerobic training [12]. Numerous evidence-based studies suggest that physical activity not only enhances physical ability and healthy conditions but also reduces mortality and serves as a preventive strategy against gerontological diseases in frail adults [13–15].

Moreover, SB and adverse health outcomes such as dementia, metabolic syndrome, obesity, and all-cause mortality among older adults has been reported [16, 17], there is a growing demand for replacing SB with physical activity in this population. In this context, replacement approaches like the Isotemporal Substitution Model (ISM) and the 24-Hour Composition Study (24-HCM) can be an effective methodological attempt [18, 19]. In this research, we focused on the ISM which is initially inspired by nutritional methodology and now widely used in the field of physical activity to observe the association between improved health outcomes and the substitution of SB with physical activity. By incorporating the total variable sum of physical activity and SB, each component is sequentially removed from the regression equation to capture the replacement effect. Previous studies on replacing sedentary behavior with physical activity in frail populations have found this replacement to be beneficial [20, 21].

Since residential areas serve as the primary mobility zones for older adults [22], the neighborhood environment plays a crucial role in supporting their daily living and physical activity. Previous studies have established a relationship between meeting physical activity guidelines and neighborhood environments [23] and highlighted the importance of the neighborhood environment on health outcomes and functional abilities in older adults [24]. Recent research has also demonstrated a significant association between walking environments and frailty prevalence [2]. However, there is limited research on how physical activity can replace SB within the context of the surrounding environment for frail older adults.

This approach is also align with one of the goal of healthy aging which supported by the action areas of the WHO Decade of Healthy Ageing, that aims to build age-friendly societies through the interaction between individuals and their environments [25–27]. Creating an age-friendly neighborhood environment is crucial, and this involves reducing barriers in the built environment by enhancing accessibility, aesthetics, and safety to ensure walkability [12, 25]. As walking is the most accessible form of physical activity for the older population, improving walkability is widely recommended as an effective means of promoting physical activity among older adults.

Meanwhile, frail older adults often experience limited mobility due to disabilities, functional impairments, and reduced independence, making physical activity a crucial strategy for maintaining health [28]. In this context, it is essential to evaluate the impact of physical activity within the framework of the surrounding neighborhood environment. This study investigates the association between SB, physical activity, and frailty, considering the built environment. We used the International Physical Activity Questionnaire (IPAQ) [29] and International Physical Activity Questionnaire Environment module (IPAQ-E) [30] adjusting for confounding variables.

Among the IPAQ-E questions, some domains, such as neighborhood safety and access to destinations, have been identified as risk factors for frailty [31]. However, within these domains, there are items that may be challenging for frail older adults to engage in, such as bicycle use and walking. While access to bicycle facilities is associated with increased physical activity prevalence [23], it may be limited for frail older adults. Therefore, we assessed the association between the perceived neighborhood environment, as measured by the IPAQ-E, assuming that certain domain-specific factors may be relevant and applicable to frail older adults.

Using ongoing nationwide representative frailty cohort study; Korean Frailty and Aging Cohort Study (KFACS) [32], we aim to assess the association between the perceived neighborhood environment based on the items within the IPAQ-E, hypothesizing that these items can be grouped together to enhance the understanding of the relationship between physical activity and frailty status. Second, we aim to explore the association between sedentary and physical activity behaviors, IPAQ-E factors, and frailty. Third, we examine this association using the ISM to assess the strength of the relationship between self-reported sedentary and physical activity behaviors and frail status when physical activity replaces SB.

## Methods

### Study population

**Fig. 1:**
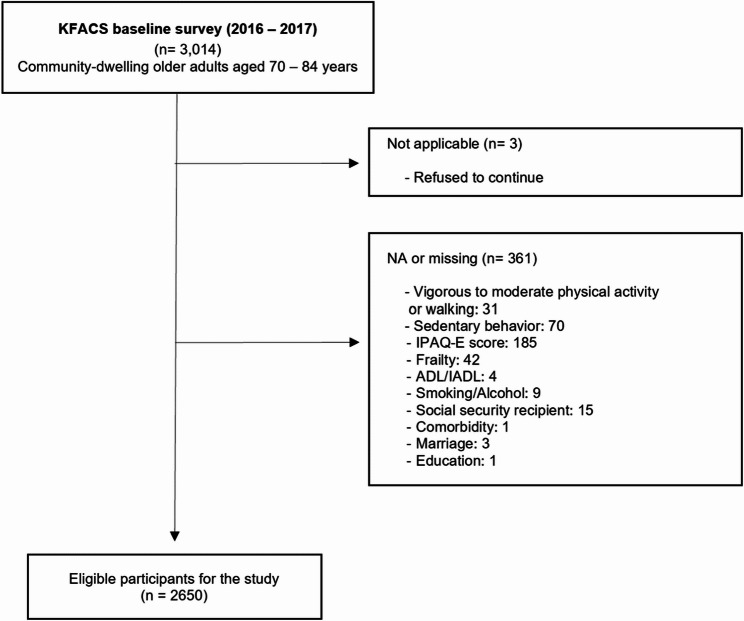
Flow gram of participation of the study

KFACS is an ongoing longitudinal cohort study with a follow-up every two years, starting with the baseline survey in 2016–2017. This cohort study was composed of sex- and age-stratified 3014 community-dwelling older adults aged 70–84 years from ten centers across South Korea [32]. The ratio for the age categories of 70–74, 75–79, and 80–84 years for enrollment was 6:5:4, and the sex ratio was 1:1. All participants in this cohort were informed about the study protocol and provided their consent, with their identities being kept anonymous. Detailed explanation of the participant recruitment methods has been introduced in the published cohort datafile [32]. The participant selection flowchart for this study is represented in Fig. 1. Among the total, 3 participants refused to continue, and 364 individuals were excluded due to missing values. The final number of eligible participants included in this research was 2650.

### Frailty

Frailty was assessed using a modified version of the Fried frailty phenotype [33], consisting of five components: weight loss, weakness, exhaustion, slowness, and low physical activity. Recent weight loss was coded as a binary variable based on the participant’s response to the question, ‘Have you experienced weight loss of more than 4.5 kg in the past year?’. Weakness was defined by the maximum handgrip strength in kilograms after measuring each hand twice using a handgrip dynamometer (T.K.K.5401; Takei Scientific Instruments Co., Tokyo, Japan). The participants were instructed to stand with their feet shoulder-width apart, arms outstretched away from the body, elbows fully extended, and shoulders and wrists in a neutral position. The participants rested for 3-min rest to ensure complete recovery after the first measurement. Exhaustion was determined based on the participant’s response to the Center for Epidemiological Studies-Depression scale (CES-D) questions [34]: ‘I felt that everything I did was an effort’ and ‘I could not get going,’ if these feelings occurred more than three days per week, then coded as a binary variable. Gait speed was measured using an automatic timer (Gaitspeedometer; Dynamicphysiology, Daejeon, Korea) over a 4-meter distance, incorporating acceleration and deceleration phases of 1.5 m each. The lowest 20% of the total population in terms of grip strength and gait speed were stratified by sex and Body Mass Index (BMI), and by sex and height, considering the nationwide distribution from KFACS, and then categorized as binary variables. Low physical activity was defined using Metabolic Equivalent Scores (METS) stratified by sex and weight based on participants’ responses to the self-reported physical activity survey, IPAQ [29]. The sum of these five domains, each coded as binary, was used to classify individuals as categorical variable following robust (score of 0), pre-frail (score of 1–2), and frail (score of 3 or more). Detailed information is provided in Table S1.

### Perceived neighborhood environment

KFACS surveyed the perceived neighborhood environment using the IPAQ-E [35], which includes 17 items assessing the built environment across 7 domains: ‘residential density,’ ‘access to destination,’ ‘infrastructure,’ ‘safety,’ ‘connectivity,’ ‘social environment,’ ‘aesthetic,’ and ‘connectivity.’ Each item has categorical response options, with responses 1–2 (strongly disagree – somewhat disagree) categorized as ‘no’ and 3–4 (somewhat agree – strongly agree) categorized as ‘yes.’ For the domains of ‘safety’ and ‘connectivity,’ the variables were re-coded in reverse. The total scores for each item were then summed by domains, with a maximum possible score of 17.

### Physical activity

The physical activity questions in KFACS are based on the IPAQ [29], which assesses the frequency and duration of different types of physical activity, such as moderate to vigorous physical activity (MVPA), walking, and SB. This self-reported survey asked participants, ‘How many days did you perform the specific activity (or SB) in a week?’ and ‘How many hours and minutes did you perform the specific activity (or SB) a day?’. For the analysis, we calculated the performance time in minutes per day by summing the minutes and hours per day following the participants’ response and then multiplying by the number of days.

### Covariates

Socio-economic status variables included age, sex, social security recipient status, marital status, BMI and education level. Age was treated as a continuous variable, while sex, social security recipient status, and marital status were regarded as binary variables. A social security recipient was defined as ‘yes’ for individuals receiving the basic national pension for the poor or medical aid. Marital status was categorized as without a spouse for those never married or widowed. BMI was grouped into low (< 18.5 kg/m^2^), normal (18.5–24.9 kg/m^2^), overweight (25–29.9 kg/m^2^) and obese (≥ 30.0 kg/m^2^). Education level was classified into ‘Not educated,’ ‘Elementary school,’ ‘Middle school,’ ‘High school,’ and ‘College or University.’ Health behaviors, including smoking and alcohol consumption, were categorized based on respondents’ reply. Health status variables included the number of comorbidities, Activities of Daily Living (ADL), Instrumental Activities of Daily Living (IADL) [36], Mini-Mental State Examination Korean version (MMSE-KC) [37]. The ADL questionnaire included items such as dressing, bathing, eating, transferring, and toileting. ADL disability was defined when one or more items were answered as ‘yes’ or ‘almost yes’. Similarly, IADL disability was defined when two or more items were ‘yes’ or ‘almost yes’ from the following list: grooming, performing household chores, cooking, doing laundry, going out, using transportation, shopping, managing money, using the telephone, and taking medication. The MMSE-KC included in the Consortium to Establish a Registry for Alzheimer’s Disease Assessment Packet (CERAD-K), is a modified version designed to address the high illiteracy rate among older adults in Korea [37] and it is a distinct version of K-MMSE (or K-MMSE-2) [38, 39]. The total MMSE-KC score was treated as a continuous variable for analysis.

### Statistical analysis

We first examined the descriptive distribution within the subject groups using the chi-square test. Then we performed factor analysis to identify combinations from the IPAQ-E items. The final number of factors was selected based on the scree plot of factor scores and loadings, with a double-check for contextual interpretation of the factor combinations. We used the varimax rotation function in the factor analysis model. For all models, we employed multinomial regression, considering the three categories of dependent variables, utilizing the ‘nnet’ package. Because the multinomial regression model computes the log odds of each category relative to the reference while simultaneously considering all categories, odds ratios (OR) can be estimated for each category (pre-frail or frail) compared to the reference group (robust) [40]. The factors were adjusted and interpreted in different ways within the model. We aimed to observe the independent association of the factors through multinomial regression analysis. Since only some of the factors were significant in the multinomial model, we further included the IPAQ-E score in the ISM analysis.

The purpose of ISM is to examine the replacement association between variables by setting the model to include each parameter and the sum of each parameter. Here, we calculated the total time (t) for physical activity and SB. Under the fixed total time (t) in the model, each variable was removed in turn, which allowed for the interpretation of the replacement of the removed variable by the remaining variables constituting the total time (t) in the model (Table S2).

Additionally, we checked the Variance Inflation Factor (VIF) of the final models to assess multicollinearity, and all variables had a VIF below the threshold (< 3) [41]. In all models, we included both physical activity and sedentary behavior (SB), allowing each variable to be adjusted for the other, thus enabling the observation of independent associations.

Lastly, sum of the IPAQ-E score and IPAQ-E factors were separately included in the models to avoid multicollinearity and analysis including IPAQ-E score has been suggested in supplementary (Table S3). We performed the subgroup analysis considering sex and age group, as suggested in the Supplementary Tables 4 & 5. Also, a sensitivity analysis was conducted on 2,498 participants after excluding 152 extreme cases of walking and MVPA (± 2.5 standard deviations [SD]) to ensure the robustness of the results (Tables S6 & S7), particularly due to the skewed distribution of physical activity (Figures S1 & S2). The threshold for statistical significance was set at *P* < 0.05, and 95% confidence intervals (CIs) were calculated for all estimates.

## Results

### Descriptive statistics of the study participants

In our population, the prevalence of each stage of frailty was 45.3% robust, 47.2% pre-frail, and 7.6% frail. Frailty was more prevalent in females (57.2%) compared to males (42.8%) and increased with age. Social security recipient status was almost twice as high in the frail group (10.9%) compared to the robust group (5.7%). The distribution of frailty was more skewed towards the lower education group (43.3%). Frail individuals had higher comorbidity rates compared to robust individuals, and the number of comorbidities increased with frailty status. Additionally, the percentage of ADL/IADL disability was higher among frail individuals, and MMSE scores worsened with frailty status. As frailty status progressed, MVPA rapidly decreased while SB increased, as shown in Figure S2. Lastly, IPAQ-E score exhibited a decreasing trend from robust to frail (Table 1).

**Table 1:** Sociodemographic and health-related characteristics by frail status

Variable			Distribution of participants (*N* = 2,650)			
			Robust **(*N*** **= 1199** (45.3%))	Pre-frail **(*N*** **= 1250** (47.2%))	Frail **(*N*** **= 201** (7.6%))	*p*-value
Sex		Male	643 (53.6)	554 (44.3)	86 (42.8)	< 0.001
		Female	556 (46.4)	696 (55.7)	115 (57.2)	
Age (mean ± SD)			75.1 (3.60)	76.4 (3.91)	78.5 (3.59)	< 0.001
Social security recipient status		Yes	69 (5.7)	95 (7.6)	22 (10.9)	0.016
		No	1130 (94.2)	1155 (92.4)	179 (89.1)	
Education level		Not educated	131 (10.9)	292 (23.4)	87 (43.3)	< 0.001
		Elementary school	277 (23.1)	361(28.9)	56 (27.9)	
		Middle school	209 (17.4)	182 (14.6)	21 (10.4)	
		High school	289 (24.1)	235 (18.8)	22 (10.9)	
		College or University	293 (24.4)	180 (14.4)	15 (7.4)	
Marriage		With spouse	322 (26.9)	439 (35.1)	80 (39.8)	< 0.001
		Without spouse	877 (73.1)	811 (64.9)	121 (60.2)	
Smoking status		Non-smoker	692 (57.7)	803 (64.2)	123 (61.2)	0.001
		Current smoker	61 (5.1)	70 (5.6)	18 (8.9)	
		Past smoker	446 (37.2)	377 (30.2)	60 (29.9)	
Alcohol consumption		Non-alcohol in a year	553 (46.1)	564 (45.1)	96 (47.8)	< 0.001
		Once to fourth/week	409 (34.1)	415 (33.2)	40 (19.9)	
		Once to fourth/month	237 (19.8)	271 (21.7)	65 (32.3)	< 0.001
Comorbidity Status		0	186 (15.5)	130 (10.4)	12 (6.0)	
		1	432 (36.0)	339 (27.1)	43 (21.4)	
		2	365 (30.4)	485 (38.8)	77 (38.3)	
		3 +	216 (18.0)	296 (23.7)	69 (34.3)	
BMI		Low	15 (1.3)	21 (1.7)	10 (5.0)	0.002
		Normal	689 (57.5)	668 (53.4)	111 (55.2)	
		Overweight	432 (36)	475 (38.0)	64 (31.8)	
		Obese	63 (5.3)	86 (6.9)	16 (8.0)	
ADL disability			11 (0.9)	26 (2.1)	18 (9.0)	< 0.001
IADL disability			271 (22.6)	292 (23.4)	77 (38.3)	< 0.001
Sum of MMSE score (mean ± SD)			26.5 (2.72)	25.2 (3.45)	23.5 (3.63)	< 0.001
MVPA (min/day)	(mean ± SD)		58.4 (84.1)	55.7 (99.6)	16.0 (37.8)	< 0.001
	(median)		28.6	14.3	0	
Walking(min/day)	(mean ± SD)		73.8 (77.5)	60.0 (76.0)	31.5 (52.9)	< 0.001
	(median)		60	30	12.9	
SB (mins/day)	(mean ± SD)		302 (154.0)	327 (174.0)	450 (238.0)	< 0.001
	(median)		300	300	420	
IPAQ-E score (mean ± SD)			13.4 (3.01)	12.4 (3.52)	11.5 (3.38)	< 0.001

*BMI* Body Mass Index, *ADL* Activities of Daily Living, *IADL* Instrumental Activities of Daily Living, *MMSE* Mini-Mental State Examination, *MVPA* Moderate to Vigorous Physical Activity, *SB* Sedentary Behavior, *IPAQ-E* International Physical Activity Questionnaire Environment module

### Multinomial regression by IPAQ-E factors

**Fig. 2:**
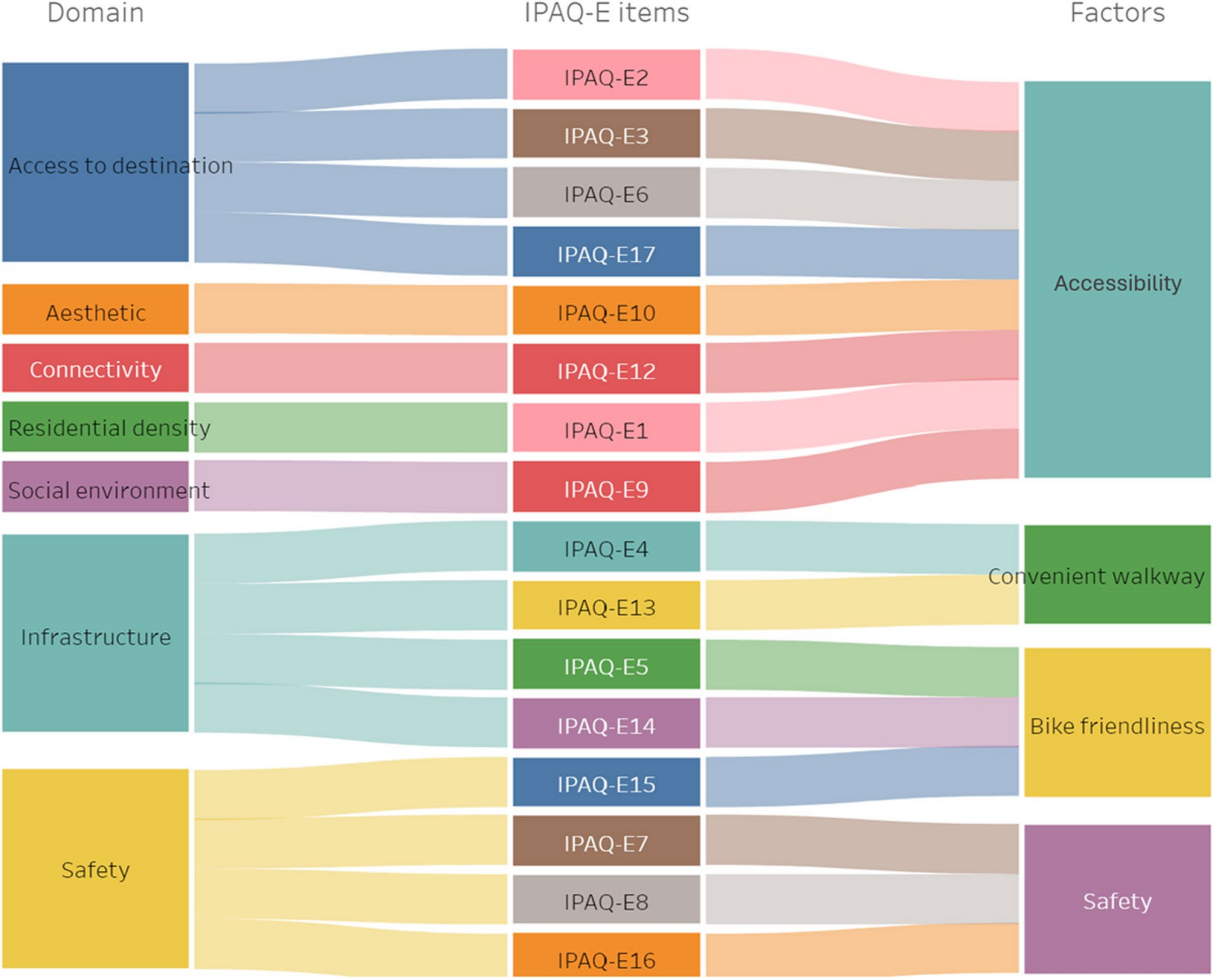
Sankey diagram for IPAQ-E items and factors

We first identified four factors from the total items in the IPAQ-E and included them in the regression equation. Each factor was named based on its shared characteristics: ‘Accessibility’, ‘Bike friendliness,’ ‘Convenient walkway,’ and ‘Safety.’ These factors were included in the models as shown in Fig. 2 and Table 2. Walking was associated with a reduced risk of pre-frailty (OR: 0.997, CI: 0.996–0.999) and frailty (OR: 0.987, CI: 0.982–0.991) when compared to robust. MVPA (OR: 0.989, CI: 0.984–0.994) was only associated with the decreased risk of frailty. Among the IPAQ-E factors, accessibility (OR: 0.750, CI: 0.673–0.836) and safety (OR: 0.861, CI: 0.769–0.965) were associated with a decreased risk of being pre-frail, while only accessibility (OR: 0.654, CI: 0.541–0.789) was significantly associated with a lower risk of frailty. The same analytical approach was applied to the model including IPAQ-E score, and the IPAQ-E score was found to be significantly associated with a reduced risk of both pre-frailty and frailty (Table S3). In the final model, which was adjusted by various covariates, IPAQ-E score was associated with the decreased risk of being pre-frail (OR: 0.936, CI: 0.910–0.962) and frail (OR: 0.915, CI: 0.870–0.963). In a subgroup analysis (Table S4), the IPAQ-E score and its factor were only significantly associated with the decreased risk of being pre-frail or frail in the age group between 70 and 79 years. In the oldest age group (over 80), only accessibility was associated with the decreased risk of being pre-frail in women (OR: 0.633, CI: 0.421–0.951).

**Table 2:** Association of self-reported sedentary and physical activity behavior, environmental factors and frailty status (*N* = 2,650)

Dependent variable	Independent variables		Model 1 OR (95% CI)	Model 2 OR (95% CI)	Model 3 OR (95% CI)
Pre-frail	MVPA (min/day)		0.999 (0.998 – 1.000)	1.000 (0.999 – 1.001)	1.000 (0.999 – 1.001)
	Walking (min/day)		**0.997 (0.996 – 0.999)**	**0.997 (0.996 – 0.999)**	**0.997 (0.996 – 0.999)**
	SB (min/day)		1.000 (1.000 – 1.001)	1.000 (1.000 – 1.001)	1.000 (1.000 – 1.001)
	Factors	Accessibility	**0.667 (0.602 – 0.739)**	**0.727 (0.653 – 0.809)**	**0.750 (0.673 – 0.836)**
		Bike friendliness	0.992 (0.897 – 1.097)	1.015 (0.916 – 1.126)	1.011 (0.911 – 1.123)
		Convenient walkway	1.028 (0.923 – 1.145)	1.021 (0.915 – 1.141)	1.012 (0.904 – 1.131)
		Safety	**0.804 (0.721 – 0.896)**	**0.86(0.776 – 0.971)**	**0.861(0.769 – 0.965)**
Frail	MVPA (min/day)		**0.985 (0.980 – 0.990)**	**0.988 (0.983 – 0.993)**	**0.989 (0.984 – 0.994)**
	Walking (min/day)		**0.986 (0.982 – 0.991)**	**0.986 (0.982 – 0.991)**	**0.987(0.982 – 0.991)**
	SB (min/day)		**1.003 (1.002 – 1.004)**	**1.002 (1.002 – 1.003)**	**1.002 (1.001 – 1.003)**
	Factors	Accessibility	**0.538 (0.453 – 0.640)**	**0.627 (0.521 – 0.753)**	**0.654 (0.541 – 0.789)**
		Bike friendliness	0.951 (0.784 – 1.153)	0.971 (0.793 – 1.189)	0.969 (0.788 – 1.192)
		Convenient walkway	1.117 (0.919 – 1.357)	1.094 (0.895 – 1.339)	1.081 (0.879 – 1.329)
		Safety	**0.740 (0.611 – 0.896)**	0.841 (0.686 – 1.031)	0.853 (0.693 – 1.051)

Bold indicates statistical significance at *P* < 0.05

Model 1 was adjusted for each of the physical activity, SB variables and IPAQ factors

Model 2 was further adjusted for sex, age, education, social security recipient, smoking, drinking alcohol and BMI

Model 3 was further adjusted for comorbidity, ADL/IADL disability and MMSE score

*MVPA*, Moderate to Vigorous Physical Activity, *SB* Sedentary Behavior, *OR* Odds Ratio, *CI* Confidence Interval

### Isotemporal substitution model

We also performed the ISM to examine the replaceable association when considering IPAQ-E score (Table 3), following the significance of result in Table S3. Substituting 10 min of SB with any type of physical activity was associated with a reduced risk of pre-frailty [if 10 min of SB was replaced by 10 min of walking (OR: 0.972, CI: 0.960–0.985)] and frailty [if 10 min of SB was replaced by MVPA (OR: 0.877, CI: 0.836–0.921); or by walking (OR: 0.852, CI: 0.814–0.891)]. Also, we could observe difference in these association by sex and age group (Table S5). In the age group between 70 and 79, walking (OR: 0.968, CI: 0.948–0.987) was associated with the decreased risk of being pre-frail only in men when substituted for SB. However, replacing SB with walking was associated with a lower risk of frailty in both men (OR: 0.859, CI: 0.787–0.938) and women (OR: 0.812, CI: 0.731–0.901). Similarly, in the oldest group age (over 80), replacing SB with MVPA (OR: 0.954, CI: 0.920–0.988) and walking (OR: 0.938, CI: 0.899–0.978) was significantly associated with the decreased risk of being pre-frail in men only. Meanwhile, substituting SB with MVPA and walking was associated with a decreased risk of being frail in both men [MVPA (OR: 0.816, CI: 0.726–0.918); walking (OR: 0.865, CI: 0.797–0.940)] and women [MVPA (OR: 0.768, CI: 0.640–0.922); walking (OR: 0.829, CI: 0.739–0.929)].

**Table 3:** Substitutional effect of SB to physical activity considering perceived neighborhood environment adjusted by IPAQ-E score (*N* = 2,650)

	(10 min/day)	SB OR (95% CI)	MVPA OR (95% CI)	Walking OR (95% CI)
Pre-frail	Substitute SB (a)	Replaced	1.002 (0.991–1.013)	**0.972 (0.960–0.985)**
	Substitute MVPA (b)	0.998 (0.988–1.009)	Replaced	**0.970 (0.955–0.986)**
	Substitute walking (c)	**1.028 (1.016–1.042)**	**1.030 (1.014–1.047)**	Replaced
Frail	Substitute SB (a)	Replaced	**0.877 (0.836–0.921)**	**0.852 (0.814–0.891)**
	Substitute MVPA (b)	**1.140 (1.086–1.197)**	Replaced	0.971 (0.909–1.037)
	Substitute walking (c)	**1.174 (1.122–1.228)**	1.030 (0.964–1.100)	Replaced

Bold indicates statistical significance at *P* < 0.05

Adjusted for sex, age, education, social security recipient status, smoking, drinking alcohol, BMI, comorbidity, ADL/IADL disability (%), MMSE score, IPAQ-E score, each of the physical activities and SB variable

OR (CI) of each row (SB, MVPA, walking) shows the results when substituted by each (a), (b), and (c)

*MVPA* Moderate to Vigorous Physical Activity, *SB* Sedentary Behavior, *OR* Odds Ratio, *CI* Confidence Interval

## Discussion

We observed an association between physical activity, IPAQ-E factors, and frailty. In the multinomial analysis, the accessibility factor was associated with a lower risk of being frail, while both accessibility and safety factors were significantly associated with a lower risk of being pre-frail, compared to the robust group. In ISM analysis, the replacement of SB by walking was significantly associated with a decreased risk of being pre-frail and frail. Replacement of SB by moderate to vigorous physical activity was significantly associated with a decreased risk of being frail.

Our results highlight that both MVPA and walking can be effective preventive strategies for frailty. This is in line with previous studies; for example, one study found that after a moderate-intensity comprehensive physical activity intervention, frailty was reversed at follow-up [42]. Another study found that multicomponent exercise has effectively improved frailty status and functionality [14]. Additionally, in our analysis using a substitutional approach, both MVPA and walking were significantly associated with a decreased risk of frailty, which is consistent with previous research [21, 43].

Interestingly, the ISM analysis showed that substituting walking with MVPA was associated with an increased risk of pre-frailty, which appears counterintuitive given the higher intensity of MVPA. One plausible explanation is that the inclusion of participants with extremely high or low physical activity levels in our dataset might have contributed to the finding that only light physical activity, such as walking, appeared to be effective in reducing the risk in the pre-frail group. Supporting this, the sensitivity analysis excluding these extreme cases demonstrated that physical activity at all intensity levels had a generally beneficial effect on both pre-frail and frail groups (Table S7). Additionally, when walking was replaced with MVPA in the model, the previously observed increased risk for the pre-frail group was no longer statistically significant. Also, no significant association was observed when MVPA was substituted with walking for frailty. This result may be attributed to the reduced sample size inherent to multinomial regression, as a large proportion of older adults reported no engagement in physical activity (Figure S1 & S2), or to potential discrepancies in self-reported physical activity measurements.

Physical inactivity is a known modifiable risk factor for frailty [44, 45]. It leads to declined energy availability by reducing the release of reactive oxygen species (ROS), resulting in muscle dysfunction [46, 47]. Considering that the frailty status can be both progressive and reversible, and in line with current physical activity guidelines [10], it is necessary to maintain physical activity.

Moreover, improving the built environment is a crucial element in constructing an age-friendly environment, as it enhances the physical activity performance [23] and is directly associated with numerous health outcomes [13, 15, 24]. However, despite the importance of physical activity interventions for active aging, challenges remain in environmental motivation research, setting environments for older adults with disabilities, and addressing societal gaps through related policy implementation [48, 49]. Therefore, we performed factor analysis with the hypothesis that there might be independently grouped environmental domains that are beneficial to frail adults and more applicable for policy adjustments. This goes beyond the already established domains from IPAQ-E, which includes items in the same domain that may be difficult for frail older adults to engage in, such as bicycle use and walking.

Although the IPAQ-E is primarily focused on the concept of walkability, its items can be re-grouped into domains through factor analysis. In our analysis, the accessibility, including transportation, destinations for purposes, recreation facilities, and aesthetic items based on mobility accessibility—were important environmental characteristics for older adults with frailty. These results are consistent with previous studies. One mixed-setting study showed an association between core items designated as accessibility and higher walking levels [50]. Additionally, 30 min of daily walking mediated the association between accessibility and aesthetics, reducing the onset of frailty [51]. Another study demonstrated an inverse association between street connectivity, walking and cycling facilities, aesthetics, and safety with frailty [52].

For older adults, the residential area is especially important as the primary area for mobility [22]. In this regard, neighborhood environment can be a crucial factor for promoting healthy lifestyle behaviors. From a public health perspective, it is necessary to enhance age-friendly neighborhood environments by reducing physical, social, and environmental barriers, taking demographic shifts into account. According to our results, the importance of accessibility is evident. Measures such as providing low-floor buses and subway entrances, placing frequently used facilities like hospitals, pharmacies, and supermarkets close to residential areas, can be implemented. Additionally, ensuring flat and obstacle-free sidewalks can facilitate safe walking to destinations. Improving parks by adding walking paths, benches, and lighting can make them more accessible for older adults.

According to our subgroup analysis, the oldest age group (over 80) still showed a protective association of physical activity and frailty in both men and women (Tables S4 & S5). Although frail adults may find it challenging to meet the guidelines, recommending these activities is still meaningful for functional improvement. Frail adults can gain substantial health benefits, such as improved physical function for independent daily living, from an active lifestyle [53]. This evidence is supported by previous research that suggests the positive effects of resistance training [54] and multicomponent individualized physical exercise on frailty [55]. Additionally, it was reported that the risk of harm from physical activity in the older adult population was very low [56].

We also need to acknowledge the limitations of our study. First, self-reported physical activity and SB questionnaires are subject to reporting or recall bias. Second, the IPAQ does not distinguish between the purposes of physical activity, such as physical activity for work, recreation, or transportation. As a result, we could not clearly capture the association between the purpose of physical activity and frailty. Third, causality cannot be established due to the cross-sectional study design. In particular, the physical activity of older populations is prone to reverse causation issues, considering their impaired health conditions such as multimorbidity. However, we attempted to address these issues by using robust models adjusting for related variables and by performing sensitivity analysis excluding participants who reported extremely low physical activity performance. In addition, the neighborhood environment can contribute to avoid reverse causation issues unless the built environment has specifically evolved to become more friendly to non-frail individuals; however, we do not have data on this aspect. Future studies should address this issue by considering the duration of residence and required to address the association of physical activity and frailty progression considering built environment using longitudinal study design.

## Conclusion

To the best of our knowledge, this was the first substitution study examining the replacement of SB with physical activity in frailty status, while considering the perceived neighborhood environment. Both physical activity and perceived neighborhood environment were independently associated with frailty status. We extracted accessibility from the IPAQ-E and observed a significant substitutional association of replacing SB with walking on frailty in both pre-frail and frail groups. We recommend enhancing an environment to support older adults’ active participation, considering the identified factors.

### Author contribution

HK designed the study concept, performed the analysis, and drafted the manuscript. CW reviewed the manuscript. SI and MJ provided insight during study design and edited the manuscript.

## Supplementary Information

Supplementary Material 1.

## Data Availability

The datasets analyzed during the current study are available upon request to the KFACS board.
