# Supplementary material for: Association between perceived neighborhood environment, sedentary behavior, walking, and moderate-to-vigorous physical activity and frailty: an isotemporal substitution model
Source: BMC Geriatr. 2025 Jul 29;25:558. doi: 10.1186/s12877-025-06200-4 (PMC12309208; doi:10.1186/s12877-025-06200-4)

**Supplementary**

**Supplementary Table 1. Frailty assessment**

|  | Men | Women |
| --- | --- | --- |
| Low grip strength | Lowest 20% of grip strength was stratified by sex and BMI based on KFACS data after measuring grip strength twice for each hands utilizing hand grip dynamometer (T.K.K.5401; Takei Scientific Instruments Co, Tokyo, Japan)). | |
|  | - BMI < 22.0, Handgrip ≤ 25.4 kg  - 22.0 ≤ BMI <23.9, Handgrip ≤ 27.1 kg  - 24.0 ≤ BMI <25.9, Handgrip ≤ 27.8 kg  - BMI ≥ 26.0, Handgrip ≤28.5 kg | - BMI < 23.0, Handgrip ≤ 16.8 kg  - 23.0 ≤ BMI <24.9, Handgrip ≤ 17.6 kg  - 25.0 ≤ BMI <26.9, Handgrip ≤ 17.8 kg  - BMI ≥ 27.0, Handgrip ≤17.7 kg |
| Low physical activity | Lowest 20% of total energy expenditure calculated by metabolic equivalent scores which derived from vigorous, moderate, and mild activities listed in IPAQ. | |
|  | < 494.65 kcal | < 283.50 kcal |
| Low gait speed | Lowest 20% of gait speed was stratified by sex and height based on KFACS data after measuring walking speed twice over 4 meters with 1.5-meter acceleration and deceleration phases utilizing automatic timer (Gaitspeedometer; Dynamicphysiology, Daejeon, Korea) and the mean values were used. | |
|  | - Height ≤ 165.0 cm, Gait speed ≤ 0.93 m/s  - Height >165.0 cm, Gait speed < 0.98 m/s | - Height ≤ 152.0 cm, Gait speed ≤ 0.85 m/s  - Height >152.0 cm, Gait speed < 0.93 m/s |
| Recent wight loss | In the last year, have you lost more than 4.5 kg unintentionally? | |
| Exhaustion | Response ‘Yes’ to 3 or more days per week for the questions; ‘I felt that everything I did was an effort’ and ‘I could not get going’. | |

**Supplementary Table 2. ISM equation**

$Total behaviors= Moderate to Vigorous physical activity+Walking+Sedantary behavior$

$$Frailty= \beta_{0}+\beta_{1}MVPA+\beta_{2}\mathrm{Walking} +\beta_{3} Total behaviors$$

(Replacement of sedentary behavior to each remainder)

$$Frailty= \beta_{0}+\beta_{1}MVPA+ \beta_{2} SB {+ \beta}_{3} Total behaviors$$

(Replacement of walking to each remainder)

$$\mathrm{Frailty}\mathbf{=} \beta_{0}+\beta_{1}\mathrm{Walking}+ \beta_{2} Sedantary behavior {+ \beta}_{3} Total behaviors$$

(Replacement of moderate to vigorous physical activity to each remainder)

**Supplementary Table 3. Multinomial regression by IPAQ-E score**

| **Dependent variable** | **Independent variables** | **Model 1**  OR (95 % CI) | **Model 2**  OR (95 % CI) | **Model 3**  OR (95 % CI) |
| --- | --- | --- | --- | --- |
| Pre-frail | MVPA (min/day) | 1.000 (0.999 – 1.001) | 1.000 (0.999 – 1.001) | 1.000 (0.999 – 1.00`) |
|  | Walking (min/day) | **0.997 (0.996 – 0.998)** | **0.997 (0.996 – 0.999)** | **0.997 (0.996 – 0.998)** |
|  | SB (min/day) | 1.001 (1.000 – 1.001) | 1.000 (1.000 – 1.001) | 1.000 (1.000 – 1.001) |
|  | IPAQ-E score (Total) | **0.906 (0.883 – 0.929)** | **0.933 (0.908 – 0.958)** | **0.936 (0.910 – 0.962)** |
| Frail | MVPA (min/day) | **0.986 (0.981 – 0.991)** | **0.989 (0.984 – 0.993)** | **0.989 (0.985 – 0.994)** |
|  | Walking (min/day) | **0.986 (0.982 – 0.990)** | **0.986 (0.982 – 0.990)** | **0.986 (0.982 – 0.991)** |
|  | SB (min/day) | **1.003 (1.002 – 1.004)** | **1.003 (1.002 – 1.003)** | **1.002 (1.001 – 1.003)** |
|  | IPAQ-E score (Total) | **0.864 (0.825 – 0.906)** | **0.909 (0.864 – 0.955)** | **0.915 (0.870 – 0.963)** |

- Bold indicates statistical significance at P < 0.05.

- Model 1 was adjusted for each of the physical activity, SB and IPAQ-E scores.

- Model 2 was further adjusted for sex, age, education, social security recipient, smoking, drinking alcohol and BMI.

- Model 3 was further adjusted for comorbidity, ADL/IADL disability and MMSE score.

- MVPA, Moderate to Vigorous Physical Activity; SB, Sedentary Behavior; IPAQ-E, International Physical Activity Questionnaire Environment module; OR, Odds Ratio; CI, Confidence Interval;

| **Dependent variable** | **Independent**  **variables** | | **Age group 1 (70 – 79)** | | **Age group 2 (80+)** | |
| --- | --- | --- | --- | --- | --- | --- |
|  |  |  | **Men**  OR (95 % CI) | **Women**  OR (95 % CI) | **Men**  OR (95 % CI) | **Women**  OR (95 % CI) |
| Pre-frail | MVPA (min/day) | | 1.001 (0.999 – 1.002) | 1.000 (0.999 – 1.002) | 0.998 (0.995 – 1.002) | 0.997 (0.992 – 1.002) |
|  | Walking (min/day) | | **0.997 (0.995 – 0.999)** | 0.998 (0.996 – 1.000) | 0.996 (0.992 – 1.000) | 1.000 (0.995 – 1.005) |
|  | SB (min/day) | | 1.000 (0.999 - 1.001) | 0.999 (0.999 – 1.000) | **1.003 (1.001 – 1.005)** | 1.001 (0.999 – 1.003) |
|  | Factors | Accessibility | **0.724 (0.609 – 0.860)** | **0.731 (0.615 – 0.868)** | 1.149 (0.791 – 1.669) | **0.633 (0.421 – 0.951)** |
|  |  | Bike  friendliness | 1.094 (0.914 – 1.311) | 0.983 (0.839 – 1.152) | 0.891 (0.628 – 1.264) | 1.145 (0.792 – 1.655) |
|  |  | Convenient  walkway | 0.936 (0.784 – 1.117) | 1.117 (0.933 – 1.338) | 0.752 (0.500 – 1.131) | 1.089 (0.743 – 1.598) |
|  |  | Safety | 0.877 (0.725 – 1.061) | 0.863 (0.723 – 1.031) | 0.764 (0.520 – 1.121) | 0.747 (0.508 – 1.098) |
| Frail | MVPA (min/day) | | **0.989 (0.981 – 0.998)** | 0.995 (0.988 – 1.002) | **0.983 (0.971 – 0.995)** | **0.978 (0.960 – 0.996)** |
|  | Walking (min/day) | | **0.988 (0.979 – 0.996)** | **0.982 (0.972 - 0.992)** | **0.990 (0.982 – 0.998)** | **0.983 (0.972 – 0.995)** |
|  | SB (min/day) | | 1.002 (1.000 – 1.004) | 1.002 (1.000 – 1.003) | **1.005 (1.002 – 1.008)** | 1.003 (1.000 – 1.005) |
|  | Factors | Accessibility | **0.500 (0.336 – 0.744)** | **0.553 (0.407 – 0.752)** | 0.945 (0.562 – 1.589) | 0.804 (0.465 – 1.391) |
|  |  | Bike friendliness | 0.714 (0.457 – 1.117) | 0.999 (0.698 – 1.429) | 0.991 (0.575 – 1.708) | 1.196 (0.714 – 2.005) |
|  |  | Convenient  walkway | 0.960 (0.634 – 1.454) | 1.000 (0.730 – 1.370) | 1.551 (0.774 – 3.108) | 1.151 (0.667 – 1.986) |
|  |  | Safety | 1.011 (0.625 – 1.636) | 0.814 (0.572 – 1.157) | 0.747 (0.428 – 1.304) | 0.722 (0.426 – 1.223) |

**Supplementary Table 4. Subgroup multinomial analysis for men and women by age group**

| **Dependent variable** | **Independent**  **variables** | **Age group 1 (70 – 79)** | | **Age group 2 (80+)** | |
| --- | --- | --- | --- | --- | --- |
|  |  | **Men**  OR (95 % CI) | **Women**  OR (95 % CI) | **Men**  OR (95 % CI) | **Women**  OR (95 % CI) |
| Pre-frail | MVPA (min/day) | **1.001 (1.000 – 1.002)** | 1.001 (0.999 – 1.003) | 0.998 (0.995 – 1.001) | 0.997 (0.993 – 1.001) |
|  | Walking (min/day) | **0.997 (0.995 – 0.998)** | 0.998 (0.996 – 1.000) | 0.996 (0.993 – 1.000) | 1.000 (0.995 – 1.005) |
|  | SB (min/day) | 1.000 (0.999 - 1.001) | 1.000 (0.999 – 1.000) | **1.003 (1.001 – 1.005)** | 1.001 (0.999 – 1.003) |
|  | IPAQ-E score | **0.926 (0.884 – 0.970)** | **0.943 (0.904 – 0.984)** | 0.945 (0.864 – 1.035) | 0.907 (0.821 – 1.003) |
| Frail | MVPA (min/day) | **0.990 (0.982 – 0.998)** | 0.996 (0.989 – 1.003) | **0.985 (0.974 – 0.996)** | **0.977 (0.960 – 0.995)** |
|  | Walking (min/day) | **0.987 (0.979 – 0.996)** | **0.981 (0.971 - 0.992)** | **0.991 (0.983 – 0.998)** | **0.984 (0.973 – 0.995)** |
|  | SB (min/day) | **1.002 (1.001 – 1.004)** | **1.002 (1.001 – 1.003)** | **1.005 (1.002 – 1.008)** | **1.003 (1.001 – 1.005)** |
|  | IPAQ-E score | **0.841 (0.758 – 0.934)** | **0.881 (0.810 – 0.958)** | 0.987 (0.861 – 1.131) | 0.958 (0.834 – 1.100) |

- Bold indicates statistical significance at P < 0.05.

- All model was adjusted by sex, age, education, social security recipient, smoking, drinking alcohol, BMI, comorbidity, ADL/IADL disability, MMSE score, each of the physical activities and SB variable.

- MVPA, Moderate to Vigorous Physical Activity; SB, Sedentary Behavior; OR, Odds Ratio; CI, Confidence Interval; IPAQ-E, International Physical Activity Questionnaire Environment module.

**Supplementary Table 5. Subgroup ISM analysis for men and women by age group**

**1. Men - Age group 1 (70 – 79)**

|  | **(10 mins/day)** | **SB**  OR (95 % CI) | **MVPA**  OR (95 % CI) | **Walking**  OR (95 % CI) |
| --- | --- | --- | --- | --- |
| Pre-frail | Substitute SB (a) | Replaced | 1.010 (0.995 – 1.026) | **0.968 (0.948 – 0.987)** |
|  | Substitute MVPA (b) | 0.990 (0.975 – 1.005) | Replaced | **0.958 (0.934 – 0.983)** |
|  | Substitute walking (c) | **1.034 (1.013 – 1.055)** | **1.044 (1.018 – 1.071)** | Replaced |
| Frail | Substitute SB (a) | Replaced | **0.887 (0.817 – 0.962)** | **0.859 (0.787 – 0.938)** |
|  | Substitute MVPA (b) | **1.128 (1.039 – 1.224)** | Replaced | 0.969 (0.856 – 1.096) |
|  | Substitute walking (c) | **1.164 (1.066 – 1.271)** | 1.032 (0.912 – 1.168) | Replaced |

**2. Men - Age group 2 (80 +)**

|  | **(10 mins/day)** | **SB**  OR (95 % CI) | **MVPA**  OR (95 % CI) | **Walking**  OR (95 % CI) |
| --- | --- | --- | --- | --- |
| Pre-frail | Substitute SB (a) | Replaced | **0.954 (0.920 – 0.988)** | **0.938 (0.899 – 0.978)** |
|  | Substitute MVPA (b) | **1.049 (1.012 – 1.087)** | Replaced | 0.984 (0.937 – 1.033) |
|  | Substitute walking (c) | **1.066 (1.022 – 1.112)** | 1.017 (0.968 – 1.068) | Replaced |
| Frail | Substitute SB (a) | Replaced | **0.816 (0.726 – 0.918)** | **0.865 (0.797 – 0.940)** |
|  | Substitute MVPA (b) | **1.225 (1.090 – 1.377)** | Replaced | 1.060 (0.927 – 1.211) |
|  | Substitute walking (c) | **1.156 (1.064 – 1.255)** | 0.944 (0.826 – 1.079) | Replaced |

**3. Women - Age group 1 (70 – 79)**

|  | **(10 mins/day)** | **SB**  OR (95 % CI) | **MVPA**  OR (95 % CI) | **Walking**  OR (95 % CI) |
| --- | --- | --- | --- | --- |
| Pre-frail | Substitute SB (a) | Replaced | 1.011 (0.993 – 1.031) | 0.985 (0.965 – 1.006) |
|  | Substitute MVPA (b) | 0.989 (0.970 - 1.008) | Replaced | 0.974 (0.948 – 1.001) |
|  | Substitute walking (c) | 1.015 (0.994 – 1.036) | 1.027 (0.999 – 1.055) | Replaced |
| Frail | Substitute SB (a) | Replaced | 0.940 (0.876 – 1.010) | **0.812 (0.731 – 0.901)** |
|  | Substitute MVPA (b) | 1.063 (0.990 – 1.142) | Replaced | **0.863 (0.758 – 0.984)** |
|  | Substitute walking (c) | 1.232 (1.110 – 1.367) | 1.158 (1.016 – 1.320) | Replaced |

**4. Women - Age group 2 (80 +)**

|  | **(10 mins/day)** | **SB**  OR (95 % CI) | **MVPA**  OR (95 % CI) | **Walking**  OR (95 % CI) |
| --- | --- | --- | --- | --- |
| Pre-frail | Substitute SB (a) | Replaced | 0.959 (0.916 – 1.005) | 0.990 (0.941 – 1.042) |
|  | Substitute MVPA (b) | 1.042 (0.995 – 1.092) | Replaced | 1.032 (0.965 - 1.105) |
|  | Substitute walking (c) | 1.010 (0.959 – 1.063) | 0.969 (0.905 – 1.037) | Replaced |
| Frail | Substitute SB (a) | Replaced | **0.768 (0.640 – 0.922)** | **0.829 (0.739 – 0.929)** |
|  | Substitute MVPA (b) | **1.302 (1.085 – 1.562)** | Replaced | 1.079 (0.870 – 1.338) |
|  | Substitute walking (c) | **1.207 (1.076 – 1.352)** | 0.927 (0.747 – 1.150) | Replaced |

- Bold indicates statistical significance at P < 0.05.

- Adjusted for sex, age, education, social security recipient status, smoking, drinking alcohol, BMI, comorbidity, ADL/IADL disability (%), MMSE score, IPAQ-E score, each of the physical activities and SB variable.

- OR (CI) of each row (SB, MVPA, walking) shows the results when substituted by each (a), (b), and (c).

- MVPA, Moderate to Vigorous Physical Activity; SB, Sedentary Behavior; OR, Odds Ratio; CI, Confidence Interval;

**Supplementary Table 6. Sensitivity analysis for older adults – multinomial analysis (N = 2,498)**

| **Dependent variable** | **Independent variables** | | OR (95 % CI) |
| --- | --- | --- | --- |
| Pre-frail | MVPA (min/day) | | **0.997 (0.995 - 0.999)** |
|  | Walking (min/day) | | **0.995 (0.993 - 0.997)** |
|  | SB (min/day) | | 1.000 (1.000 - 1.001) |
|  | Factors | Accessibility | **0.771 (0.687 - 0.865)** |
|  |  | Bike friendliness | 1.003 (0.900 - 1.118) |
|  |  | Convenient walkway | 0.969 (0.861 - 1.091) |
|  |  | Safety | **0.887 (0.788 - 0.998)** |
| Frail | MVPA (min/day) | | **0.988 (0.982 - 0.993)** |
|  | Walking (min/day) | | **0.982 (0.977 - 0.987)** |
|  | SB (min/day) | | **1.002 (1.001 - 1.003)** |
|  | Factors | Accessibility | **0.669 (0.552 - 0.812)** |
|  |  | Bike friendliness | 0.987 (0.799 - 1.219) |
|  |  | Convenient walkway | 1.054 (0.854 - 1.302) |
|  |  | Safety | 0.850 (0.688 - 1.049) |

| **Dependent variable** | **Independent variables** | OR (95 % CI) |
| --- | --- | --- |
| Pre-frail | MVPA (min/day) | **0.997 (0.996 - 0.999)** |
|  | Walking (min/day) | **0.995 (0.993 - 0.997)** |
|  | SB (min/day) | 1.000 (1.000 - 1.001) |
|  | IPAQ-E scores (Total) | **0.938 (0.911 - 0.965)** |
| Frail | MVPA (min/day) | **0.988 (0.983 - 0.993)** |
|  | Walking (min/day) | **0.982 (0.977 - 0.987)** |
|  | SB (min/day) | **1.002 (1.002 - 1.003)** |
|  | IPAQ-E scores (Total) | **0.919 (0.872 - 0.968)** |

- Bold indicates statistical significance at P < 0.05.

- The sum of the IPAQ-E score and IPAQ-E factors were separately included in the models to avoid multicollinearity.

- Adjusted for sex, age, education, social security recipient, smoking, drinking alcohol, BMI, comorbidity, ADL/IADL disability, MMSE score, each of the physical activities and SB variable.

- MVPA, Moderate to Vigorous Physical Activity; SB, Sedentary Behavior; IPAQ-E, International Physical Activity Questionnaire Environment module; OR, Odds Ratio; CI, Confidence Interval;

**Supplementary Table 7. Sensitivity analysis for older adults – ISM analysis (N = 2,498)**

|  | **(10 mins/day)** | **SB**  OR (95 % CI) | **MVPA**  OR (95 % CI) | **Walking**  OR (95 % CI) |
| --- | --- | --- | --- | --- |
| Pre-frail | Substitute SB (a) | Replaced | **0.969 (0.954 - 0.985)** | **0.948 (0.931 - 0.966)** |
|  | Substitute MVPA (b) | **1.032 (1.015 - 1.048)** | Replaced | 0.978 (0.955 - 1.002) |
|  | Substitute walking (c) | **1.054 (1.035 - 1.074)** | 1.022 (0.998 - 1.047) | Replaced |
| Frail | Substitute SB (a) | Replaced | **0.866 (0.822 - 0.913)** | **0.812 (0.771 - 0.855)** |
|  | Substitute MVPA (b) | **1.154 (1.096 - 1.216)** | Replaced | 0.937 (0.871 - 1.008) |
|  | Substitute walking (c) | **1.232 (1.170 - 1.297)** | 1.067 (0.992 - 1.148) | Replaced |

- Bold indicates statistical significance at P < 0.05.

- Adjusted for sex, age, education, social security recipient status, smoking, drinking alcohol, BMI, comorbidity, ADL/IADL disability (%), MMSE score, IPAQ-E score, each of the physical activities and SB variable.

- OR (CI) of each row (SB, MVPA, walking) shows the results when substituted by each (a), (b), and (c).

- MVPA, Moderate to Vigorous Physical Activity; SB, Sedentary Behavior; OR, Odds Ratio; CI, Confidence Interval;

**Supplementary Figure 1. Density plot of types of physical activity and sedentary behaviors by frail status**


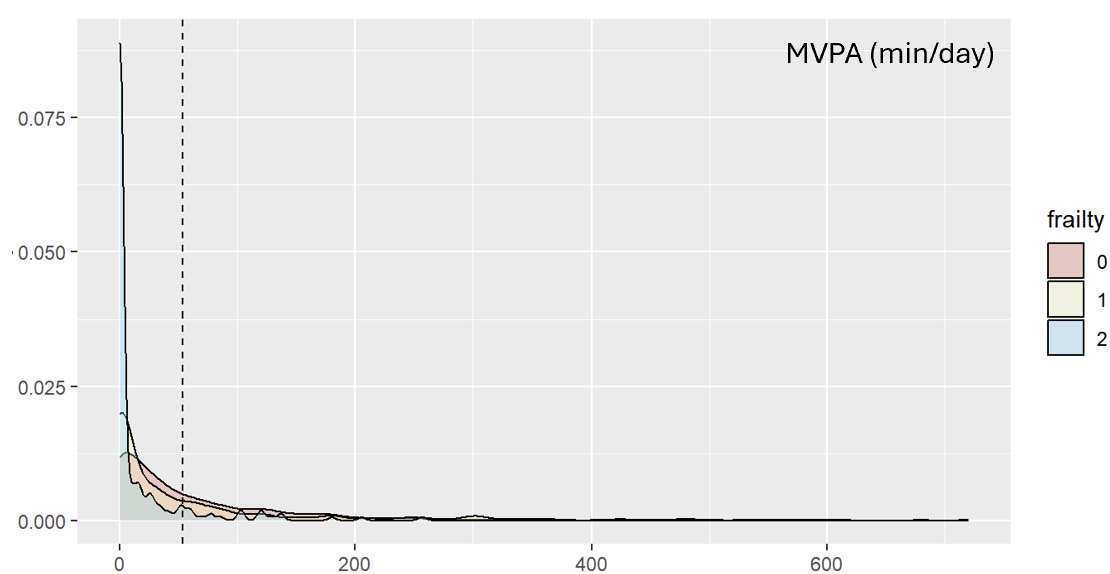


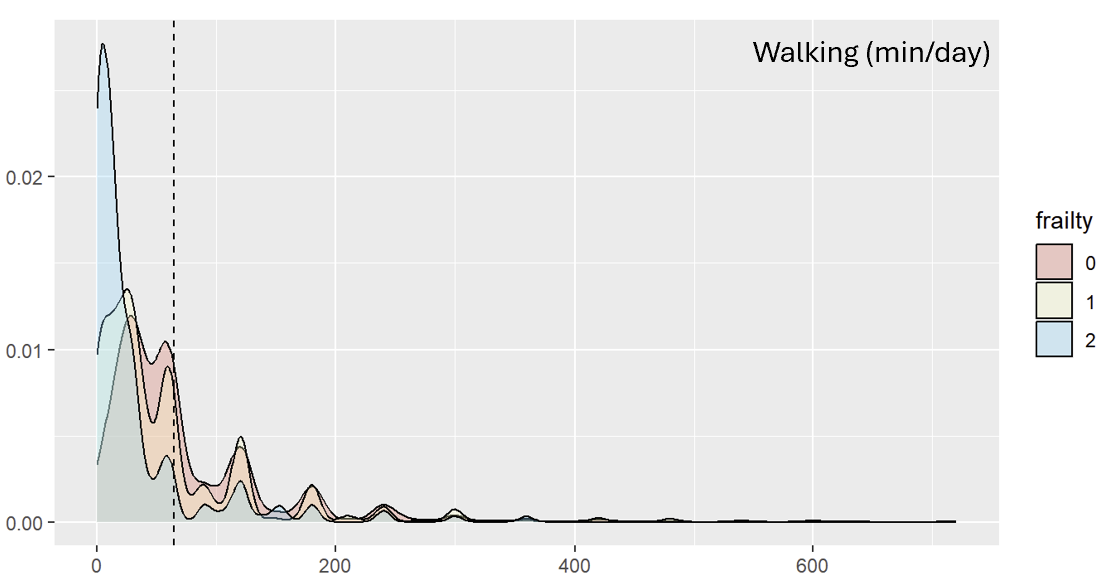

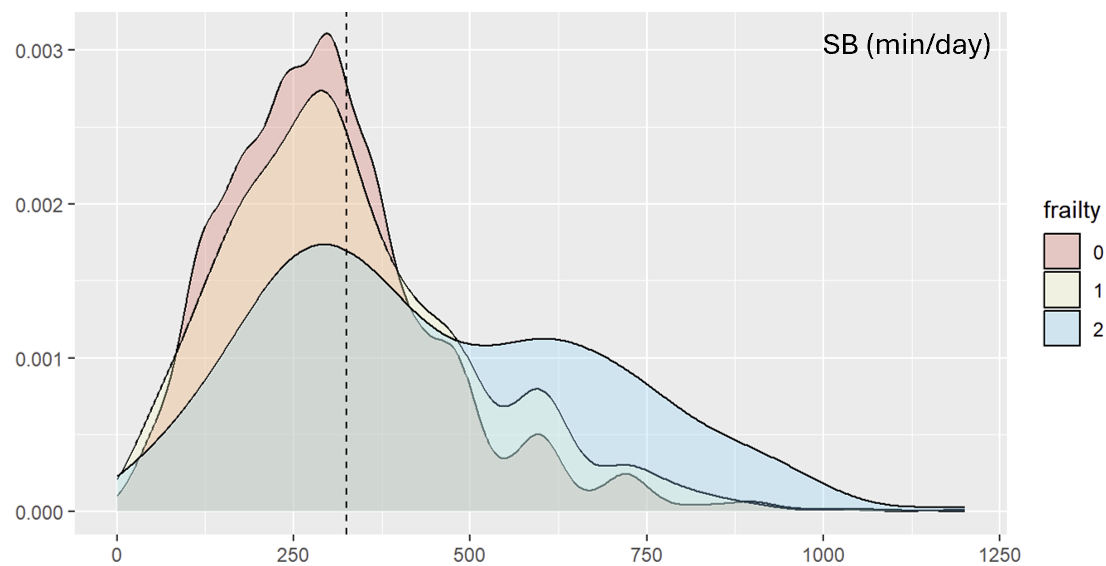


**Supplementary Figure 2. Physical activity distribution for the sensitivity analysis**


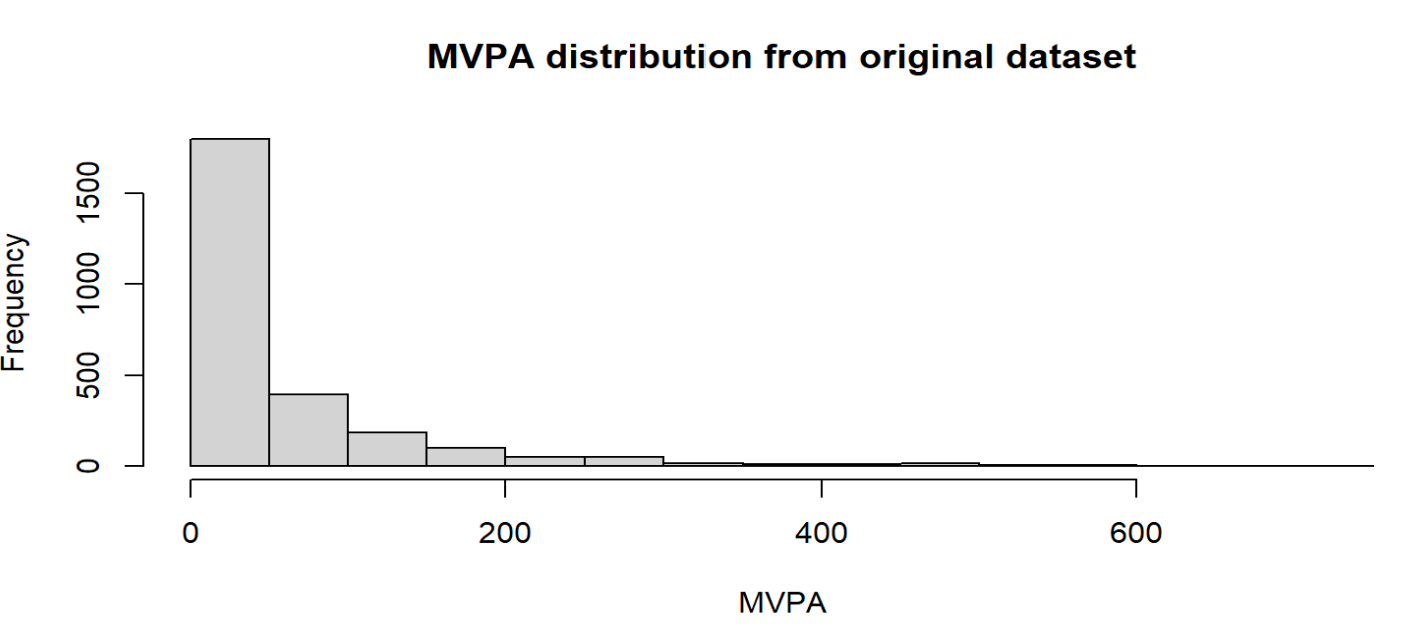


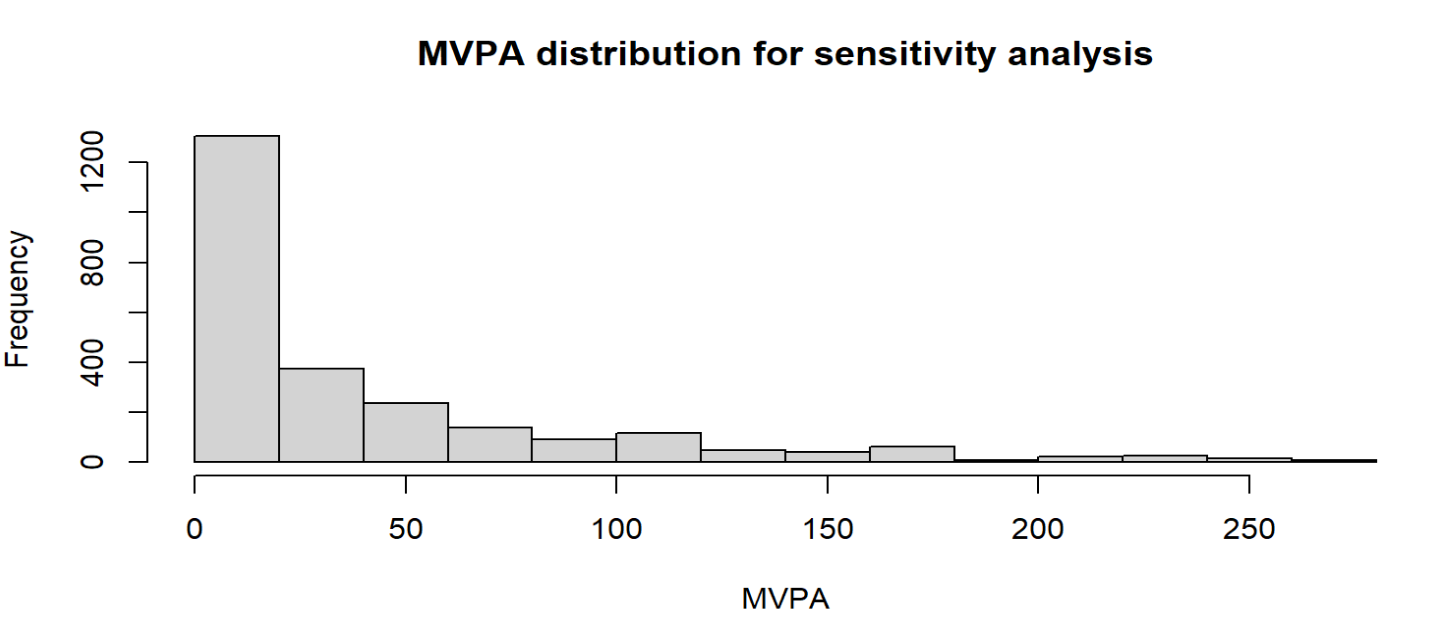


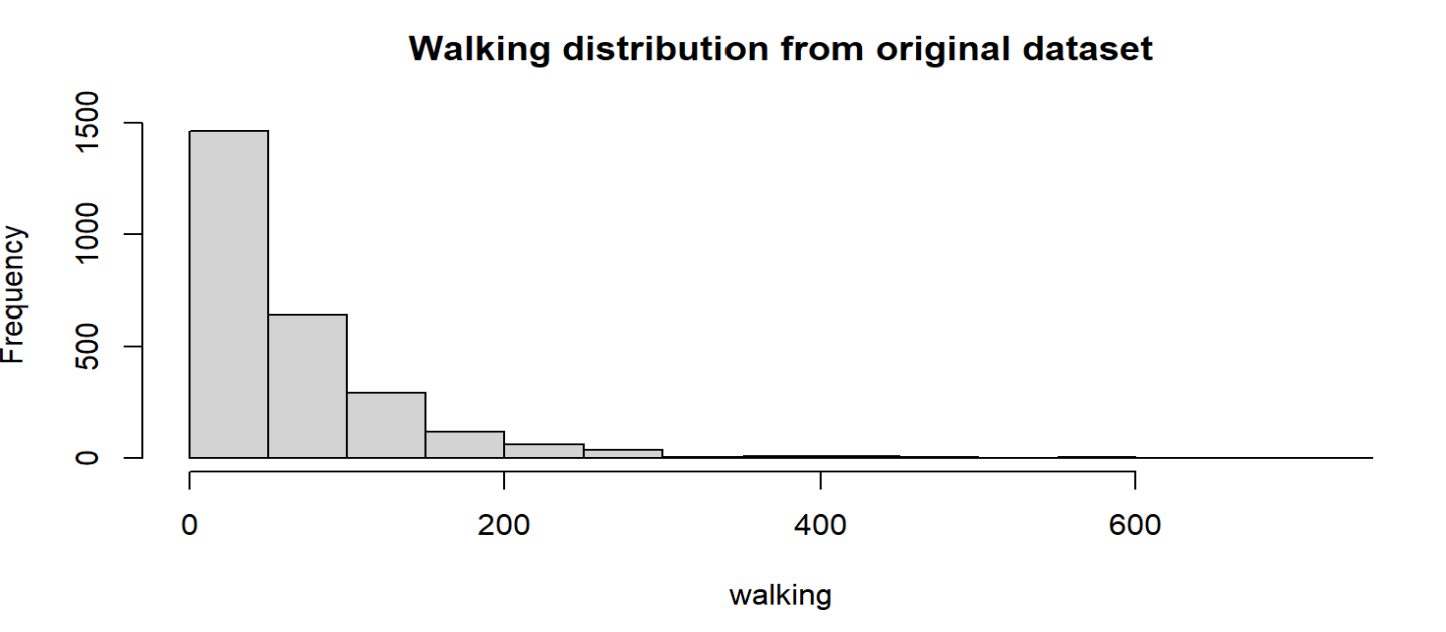


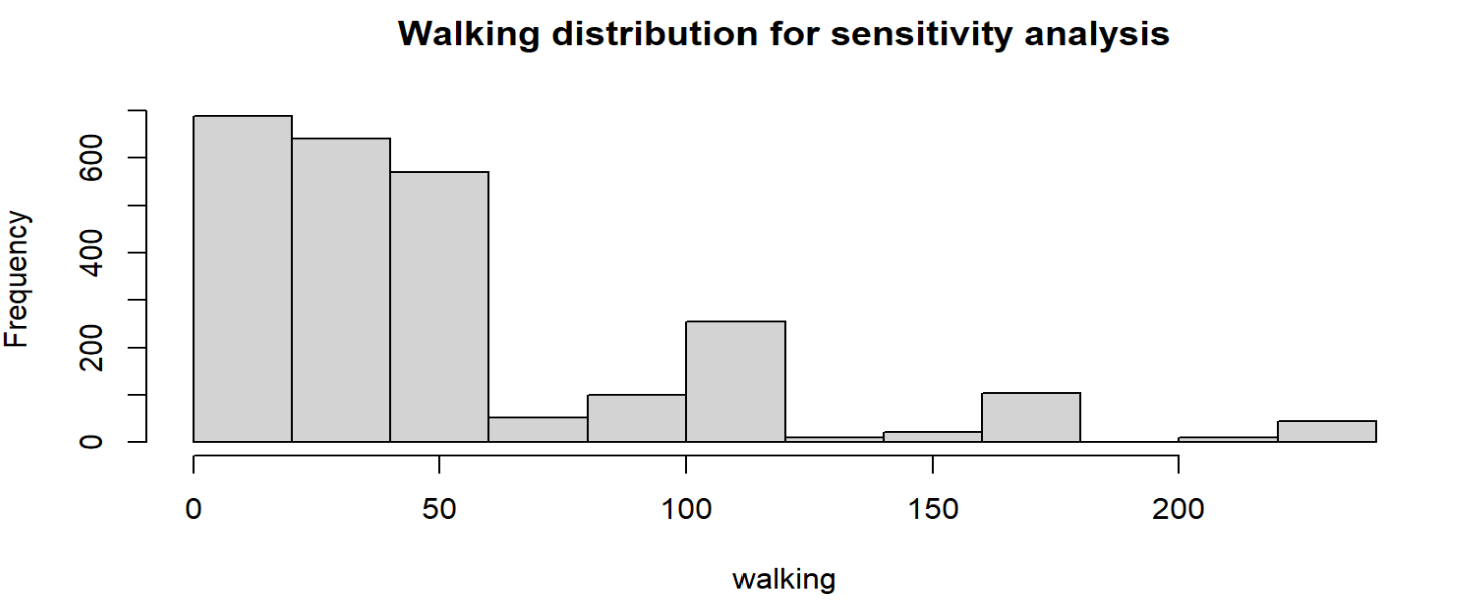

Supplement: Supplementary file 1 — Supplementary Material 1. [file 12877_2025_6200_MOESM1_ESM.docx]
